# Applying the behaviour change wheel to develop a smartphone application ‘stay-active’ to increase physical activity in women with gestational diabetes

**DOI:** 10.1186/s12884-022-04539-9

**Published:** 2022-03-26

**Authors:** R. Smith, M. Michalopoulou, H. Reid, S. Payne Riches, Y. N. Wango, Y. Kenworthy, C. Roman, M. Santos, J. E. Hirst, L. Mackillop

**Affiliations:** 1 Department of Sport and Exercise Medicine, Nuffield Orthopaedic Centre, Oxford University Hospitals NHS Trust, Windmill Road, Oxford, OX3 7LD UK; 2 Nuffield Department of Primary Care Health Sciences, University of Oxford, Oxford, England; 3 Women’s Centre, Oxford University Hospitals NHS Foundation Trust, Oxford, England; 4 Institute of Biomedical Engineering, Department of Engineering Science, University of Oxford, Oxford, England; 5 Nuffield Department of Women’s & Reproductive Health, University of Oxford, Oxford, England

## Abstract

**Background:**

Physical activity (PA) interventions are an important but underutilised component in the management of gestational diabetes mellitus (GDM). The challenge remains how to deliver cost effective PA interventions that have impact on individual behaviour. Digital technologies can support and promote PA remotely at scale. We describe the development of a behaviourally informed smartphone application (Stay-Active) for women attending an NHS GDM clinic. Stay-Active will support an existing motivational interviewing intervention to increase and maintain PA in this population.

**Methods:**

The behaviour change wheel (BCW) eight step theoretical approach was used to design the application. It provided a systematic approach to understanding the target behaviour, identifying relevant intervention functions, and specifying intervention content. The target behaviour was to increase and maintain PA. To obtain a behavioural diagnosis, qualitative evidence was combined with focus groups on the barriers and facilitators to PA in women with GDM. The findings were mapped onto the Capability Opportunity Motivation-Behaviour (COM-B) model and Theoretical Domains Framework to identify what needs to change for the target behaviour and linked to appropriate intervention functions. Finally, behaviour changes techniques (BCT) and modes of delivery that are most likely to serve the intervention functions were selected. Current evidence, patient focus groups and input from key stakeholders informed Stay-Active’s development.

**Results:**

We found that psychological capability, reflective and automatic motivation, social and physical opportunity needed to change to increase PA in women with GDM. The four key intervention functions identified were Enablement, Education, Persuasion and Training. Stay-Active incorporates these four intervention functions delivering ten BCTs including: goal setting, credible source, self-monitoring, action planning, prompts and cues. The final design of Stay-Active delivers these BCTs via an educational resource centre, with goal setting and action planning features, personalised performance feedback and individualised promotional messages.

**Conclusion:**

The BCW has enabled the systematic and comprehensive development of Stay-Active to promote PA in women with GDM within an NHS Maternity service. The next phase is to conduct a trial to assess the feasibility and acceptability of a multi-component intervention that combines Stay-Active with PA Motivational Interviewing.

**Supplementary Information:**

The online version contains supplementary material available at 10.1186/s12884-022-04539-9.

## Introduction

Gestational diabetes mellitus (GDM) is defined as any degree of glucose intolerance first detected during pregnancy [1]. It has an increasing prevalence worldwide [2]. GDM is associated with serious complications for both mother and baby [3–5]. Fundamental to the management of GDM is glycaemic control [6], with increasing levels of blood glucose suggested as the mechanism for the increased risk of adverse maternal and infant outcomes [7].

There is growing evidence indicating the benefits of physical activity (PA) amongst women with GDM. Meta-analyses of interventions to increase PA among pregnant women, have shown improvements in glycaemic control and reduced insulin requirements [8, 9]. Prescription of aerobic or resistance exercises appears to be effective, particularly those that are performed at a moderate intensity and for a minimum of three times a week [9]. Guidance on the clinical management of GDM, from the National Institute for Health and Care Excellence (NICE), recommends healthcare professionals to advise women with GDM to exercise regularly [10]. Despite this national guidance, the promotion of PA is an underutilised management tool. This pattern is reported globally, a recent report from Brazil found that at least 65% of women with GDM are not meeting PA recommendations [11] and only a small proportion (6.7.%) were using healthcare professionals as their source of information on exercise in pregnancy [12]. Qualitative reports have found that women with GDM would prefer clear, simple and specific PA messages with flexible options [13]. Face-to-face or phone follow-up can achieve higher levels of adherence to exercise interventions with support from healthcare professionals [9]. However, translating the positive research findings from PA interventions into routine care for GDM remains a challenge. This is because time allocation, staff training, and availability of resources, all compete with other components of care. As a result, many women with GDM may receive little, or no, PA advice.

Fundamental to the success of PA interventions is a sound theoretical basis with the incorporation of appropriate Behaviour Change Techniques (BCTs), particularly those that are person-centred, addressing specific barriers and enablers [14]. For example, techniques such as goal setting and action planning, shaping knowledge and comparison of outcomes have been effective in attenuating the observed decline of PA during pregnancy [15]. The diagnosis of GDM may provide a ‘teachable moment’ [16] when women are motivated to optimise their health over a short period of time and are likely to respond well to behaviour change interventions. The UK Medical Research Council (MRC) Complex Intervention Framework emphasises the requirement of using appropriate theory in intervention design [17] to improve effectiveness and allow the behaviour change components to be replicated [18]. The Behaviour Change Wheel (BCW) is an evidence-based theory with a comprehensive framework. The BCW delivers a systematic approach to understanding the target behaviour, identifying relevant intervention functions, and specifying content [18]. It has been successfully used to design other complex PA interventions [19–21].

There is increasing interest around wearable device and smartphone applications to increase PA with modest evidence for effectiveness [22]. However, to date, most apps to promote PA have been developed without the integration of appropriate BCTs [23]. A systematic review of the efficacy of interventions that use apps to improve diet, PA and reduce sedentary behaviours, highlighted several common features present in effective PA app interventions, including goal setting, self-monitoring, performance feedback, motivational messaging, and game-like features. The authors concluded that there is modest evidence that app-based interventions can be effective for increasing PA. Multi-component interventions appear to be more effective than standalone interventions [24]. Promising results from a randomised trial found that the combination of a mobile phone app and brief counselling increased objectively measured PA over 3 months in physically inactive non-pregnant women in the USA [25].

Motivational interviewing offers a method to deliver several effective BCTs. It is designed to strengthen personal motivation and commitment to individualised goals by eliciting and exploring the person’s own reasons for change, and addressing key barriers and enablers [26]. In non-pregnant populations, motivational interviewing has been shown to improve health behaviours such as reducing alcohol consumption and improving PA levels [27]. Amongst pregnant women, motivational interviewing has been effective in improving healthy eating behaviours [28–30]. No studies to date have explored motivational interviewing-based PA interventions in women with GDM. However, a recently published quality improvement project undertaken by authors of this report, where motivational interviewing was incorporated into the routine clinical care for 64 women with GDM, found a significant increase in self-reported PA levels after 2 weeks [31]. Women were invited to a 20-min individual motivational interview on PA by a trained health care professional (HCP). A specific motivational interviewing framework was used including key micro-skills, individual goal setting, activity planning and specific information about the benefits and types of suggested PA (further details within Additional file 1). Motivational interviewing may provide the initial catalyst for behaviour change. The challenge remains how to maintain and support this change. Digital technologies provide an opportunity to support and promote PA remotely and are already used for remote management of glycaemic control in this setting [32]. The smartphone application (Stay-Active) has been designed to enhance and support the existing motivational interviewing intervention. This report aims to describe the design and development, using the BCW of Stay-Active to promote and maintain PA in women attending an NHS GDM clinic.

## Methods

### Setting

Stay-Active was designed for women diagnosed with GDM at the Women’s Centre, Oxford University Hospitals NHS Foundation Trust. Women attending had a confirmed diagnosis of GDM as defined by International Association of Diabetes and Pregnancy Study Groups recommendations [33]. Within this clinical service, a PA motivational interviewing intervention was already being delivered [31] (see Additional file 1). Stay-Active was developed by specialist clinicians in this unit, to support this intervention.

### Design

**Fig. 1:**
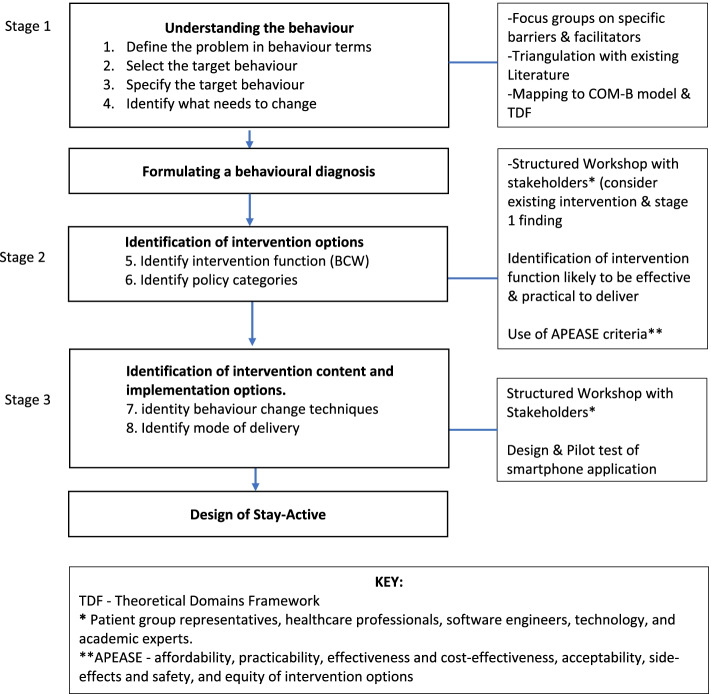
Shows a flow diagram of the method used

We applied the BCW [34] to inform the development of Stay-Active. This involved gathering evidence from current qualitative literature and focus groups. Figure 1 provides an overview of the method. In brief, the steps followed were:

- stage 1: understanding the behaviour
- stage 2: identification of intervention options
- stage 3: identification of intervention content and implementation options.

### Stage 1: understanding the behaviour

Central to the BCW, is the COM-B model. This comprises of Capability (physical and psychological), Opportunity (social and physical) and Motivation (automatic and reflective). Michie et al. [18] propose that people need these three factors to enhance the likelihood of performing the behaviour in question. The COM-B model is supported by the Theoretical Domains Framework (TDF) [35]. This framework was applied for a deeper exploration and understanding of the barriers to and facilitators of change for the target behaviour [36]. To identify the key facilitators and barriers to PA for pregnant women with GDM and inform our behavioural analysis, we reviewed and synthesised the latest relevant qualitative evidence [12, 13, 37–41] and invited women with GDM to attend focus groups as part of patient and public involvement (PPI) in line with Oxford University Hospital Trust’s PPI Strategy and Policy.

Two focus groups (total of ten women) were conducted online via the video conferencing application Microsoft Teams. Participants were invited to the focus groups at their routine outpatient appointment following an initial discussion with a specialist midwife. Participants were invited over a one-month period on a voluntary basis. They were then welcomed to attend one of two online focus groups. Participants were not offered any reimbursement. A leaflet was provided explaining the purpose of the session and they were conducted in line with the Hospital Trust’s PPI Strategy. The groups were facilitated by two trained clinical researchers (authors RS, YK & NW). The researchers guided the discussions with open-ended questions around the four topic areas but refrained from directly taking part in the discussions. Each session lasted approximately 1 h. A summary of the discussion was provided by one of the researchers at the end of each focus group with the opportunity to clarify or add any missing views. Written notes were made at each focus group. The sessions were not directly recorded or transcribed. The notes were then analysed, with themes identified and mapped onto the six components of the COM-B model.

Focus groups discussed the following topics:

1. perceptions of physical inactivity on their health
2. perceptions of the barriers to increase and maintaining PA
3. perceptions of facilitators (Capability, Opportunity and Motivation) to increase PA levels
4. Feedback was sought on potential design components of Stay-Active

This data was then used to inform the COM-B behavioural analysis. This method identified the sources of behaviour that were involved increasing PA in women with GDM and resulted in a ‘behavioural diagnosis’.

### Stage 2: identification of intervention options

To determine the intervention functions most likely to affect behaviour change, we convened a workshop with key stakeholders including patient group representatives, healthcare professionals, software engineers, technology, and academic experts. This workshop was facilitated by the researchers (authors RS & LM) and resulted in intervention functions were mapped onto the COM-B model with the behavioural diagnosis. During the workshop, the components of existing motivational interviewing intervention and the information gathered from stage 1 were presented and considered to how Stay-Active could best help to increase and maintain PA. The stakeholder groups chose from nine possible functions, including: education; persuasion; incentivisation; coercion; training; restriction; environmental restructuring; modelling and enablement. Stakeholders considered and selected these possible intervention functions using the APEASE criteria (affordability, practicability, effectiveness and cost-effectiveness, acceptability, side-effects and safety, and equity of intervention options) [18].

### Stage 3: intervention content

This stage included the identification of Behaviour Change Techniques (BCTs) (the active ingredients of the intervention) and the identification of the mode of delivery. As in stage 2, these steps were also informed discussions with stakeholders in a second workshop. From the list of 93 BCTs [42], the stakeholders agreed and selected the most appropriate BCT that would bring about the desired change (i.e. being physically active). BCTs that were already delivered during the motivational interviewing were considered and how Stay-Active could further support these; seeking to increase and maintain PA. The mode of delivery for each BCT was also selected as part of the implementation plan. The practical application and delivery of the intervention was discussed. Finally, the actual behaviour change intervention activities were identified and designed. The development team (researchers and software engineers) shared, trialed, and piloted specific design components with the PPI group.

## Results

### Stage 1: understanding the behaviour

The target behaviour identified was to increase and maintain PA for women with GDM (Table 1). Specific and generic enablers and barriers to PA for women with GDM. Several themes emerged and were linked to COM-B framework. Tables 2 and 3 summarise the barriers and enablers from the focus groups and literature.

**Table 1:** Specify target behaviour

*What target behaviour?*	Increasing and maintaining physical activity
*Who needs to perform the behaviour?*	Insufficiently active women with Gestational Diabetes Mellitus
*What does the person need to do to have the preferred outcome?*	Participate at least 150 min per week, motivation to be more active
*Who is involved in performing the behaviour?*	By themselves, or with others or groups or in group classes or family members.
*When will they perform the behaviour?*	Time convenient to them/(opportunities)
*Where will they perform the behaviour?*	Parks, walking routes, place of work, leisure centre, at home, gym

**Table 2:** Barriers and enablers to physical activity for Women with diagnosis of GDM collected from the PPI groups

COM-B component	Theme	Example of Enablers	Example of Barriers
**Psychology capability –** knowledge or psychological skills, strength or stamina to engage in the necessary mental process	Limited specific knowledge of PA benefits, types of PA in pregnancy and PA resources in GDM Safety concerns	Awareness of the types of activity to perform; reassurance activity can be started gradually Awareness of specific benefits to blood glucose control and reducing weight gain. Information from a source in which they had confidence . Instructions for the specific exercise, what type of activity *‘am I ok to do’*. Understanding the importance of activity in the treatment of GDM	So much information on the internet, difficulty knowing what to trust. Lack of confidence to start a new exercise and fear of causing harm
**Physical capability –** physical skill, strength, stamina	Pregnancy symptoms (pain, nausea, lack of energy, tiredness)	Exercise can improve symptoms ‘*I would always feel better once had been for a walk’*	*‘Had nausea in the 1st Trimester, I completely stopped doing all activity’*
**Social opportunity –** Opportunity afforded by interpersonal influences, social cues and cultural norms that influence the way we think	Partners & family support Work & childcare Support from Classes	*Social support* *‘Since the diagnosis my partner has been more supportive and is almost dragging me out for walks’* *Zoom and Facebook instruction classes were really helpful’*	Difficulties with Work, Childcare and maintaining consistency
**Physical Opportunity** – Opportunity afforded by the environment involving time, resources, location, cues physical affordance	Finding Time for activity Finding local activity Weather	Online classes and home exercises have been really helpful especially in lockdown – ‘*I am more comfortable to exercise at home and can fit in with my schedule’* Activity groups close to home	*‘Finding the time when you have look after a two-year-old all day’* *‘Exercising in daylight hours, you just don’t feel like going out for a walk in the dark’* Having the time and adapting around children
**Reflective Motivation –** Reflective process involving plans (self-conscious intentions) and evaluations (beliefs about what is good and bad)	For Health of the Baby and reducing the risk of complication Intention: GDM was a prompt to start to activity	Every pregnancy is different, depends on the person and the time in the pregnancy Baby Health and Responsibility *‘Concerns about the baby and doing everything I can’ & It isn’t about me it is about my baby’* Getting a diagnosis of GDM – has been *‘prompt for me to review my activity level’* *‘Activity is now even more important to my health since the diagnosis of GDM’* Desire to be in good shape for labour, want to be active for the future, ‘*Doing some activity made me feel better.*’	Mind-set: *‘you get into a comfortable zone and that a change to the routine will stress you out’* Habit: *‘Had a sense that I did not need to be active’*
**Automatic Motivation** – automatic processes involving emotional reactions, desires (wants and needs) impulses inhibitions drive states and reflex responses	Desire Worry and fear	Having high blood sugar levels can prompt me to do activity Worry: *‘about the health of the baby and that I am doing everything I can’* Worry of having to go onto medication	

Note: Direct quotes are from different participants

*Abbreviations*: *GDM* Gestational diabetes Mellitus, *PA* Physical activity

**Table 3:** Barriers and facilitators of physical activity (PA) in women with GDM mapped onto COM-B and TDF components & Behavioural analysis and diagnosis of the behavioural sources that contribute to physical activity (PA) in this group

COM-B component	Barriers and facilitators to PA in women with GDM^**a**^	Theoretical Domains Framework	What needs to happen for change to occur?
Psychological capability	Information is considered as important, awareness of specific benefits of PA with GDM (Both mother & Baby), (+) Information can positively influence individuals’ intentions towards maintaining PA (+) Family/partners understanding of the importance of PA affects the women’s attitudes to PA (+) Resilience to make change (+) Lack of knowledge & understanding of what counts towards PA, types of PA & location of specific resources (−) Fear over safety of activity (−) Lack of awareness of the implication of being inactive with GDM (−)	Knowledge & understanding decision making	Awareness of specific benefits from a credible source, given permission, support from partner/family Awareness what activity is safe for them
	Self- monitoring, Women expressed interest in goal setting (+)	Behaviour regulation	
Physical capability	Effects/Medical conditions of pregnancy & symptoms (nausea, fatigue) (−) Ability to perform activity due to pregnancy (high risk) concerns (−) PA can improve physical symptoms (+)	Skills	No change required - the individuals will have the exercise capacity to maintain PA
Social opportunity	Exercise based programmes from Maternity HCP is regarded as safe/supportive as they are associated with the health care system (+) Support and understanding from HCPs is important e.g. HCPs provide a sense of security/comfort (+) Maintenance of support from family and friendly is important, e.g. partner provide a sense of solidarity/support (+) Acceptability & culture of PA in pregnancy, particularly within families (e.g. overprotective) (+/) Interaction with other pregnant women Home responsibilities; caring for child or partner limits PA opportunities (−) Negative pressure/culture from family leads to avoidance of PA (−)	Social influences (Process that can change thoughts feelings or behaviours – social pressure)	Individuals confident with PA programmes Support from HCP
Physical opportunity	Lack of access to physical activity/leisure (facilitates) (−) Lack of outdoor space to be space (−) Lack of time (childcare/work commitments) (−)	Environmental context and resources (persons situation or environment)	Time, resources and location influence PA choice, affecting behaviour.
Reflective motivation	Belief about capacity ‘time for change’ particularly for the benefit of the baby’) (+) PA is associated with feeling of guilt or frustration/concerns due to reduce capacity compared to pre-pregnancy, which leads to avoidance of PA (−) Feeling responsible (+)	Beliefs about capability (acceptance of the truth, reality or validity about an ability, perceived behavioural control, self-esteem, confidence)	Self-efficacy influences approach to PA. Belief about capability toward PA Increased self-monitoring and feedback
	Recognition of improvements through self-monitoring and feedback leads individuals to recognise their capabilities and increase motivation for PA (+)	Goals	
	Belief that PA is enjoyable and leads to health benefits (+) Self-efficacy: activity may lead to harm & avoidance of PA (due to health beliefs)	Belief about consequences	
Automatic motivation	PA is associated with discomfort/pain, which leads to avoidance of PA (−) (due to negative emotions associated activity) Establishing a routine (+) and maintaining habits after (+) are important in the maintenance of PA Pre-pregnancy PA habits (+/−) Apprehensive of PA in public place (−) Fear/anxiety based on previous pregnancy/miscarriage _(−)	Emotion	Habits and routines influence behaviour

*Abbreviations*: *PA* Physical Activity, *TDF* Theoretical Domains Framework, *GDM* Gestational Diabetes, *HCP* Health care professionals

^a^Barrier and enablers drawn from both focus group and current literature [12, 13, 37–41]

### Psychological capability

Both qualitative evidence and our focus groups reported barriers surrounding women’s knowledge on the specific benefits of PA and concerns about safety. For example both source identified that women reported a lack of information on safe activities and described the information received from their midwife as ‘limited’ [43]. Enablers included knowledge of the benefits for the mothers and baby’s health [37, 38]. Women with GDM want messages to be explicit about what and how much PA they need to participate in for themselves and the health of their baby, which is a strong motivator [13]. Semi-structured interviews with women with GDM in Australia found they want clear, simple, specific PA messages directly related to pregnancy outcomes that are delivered by a credible source [13].

Our focus groups found more specific enablers related to an increased awareness of PA levels to prevent GDM complications and specific knowledge about the benefits and effects of PA related to glycaemic control.

### Physical capability

Pregnancy symptoms/discomforts (particularly fatigue and nausea) and experiencing pain were highlighted in the focus groups and current literature as common barriers that prevent women from being active [43].

### Physical opportunity

Lack of time, access to facilities and weather were identified as a common barriers across the literature [14], including having other children and working [43].

Our focus group discussions highlighted online and home exercise classes as enablers with participants emphasising the importance of being able to fit PA around their schedules.

*‘I am more comfortable to exercise at home and can fit in with my schedule’*

### Social opportunity

Social support is a key enabler [37, 38].Women suggested being active was easier when supported by their partners, family, or friends. Furthermore, support and understanding from HCPs provided a sense of security and comfort.

Focus group participants emphasised the importance of support from partners but also online social media groups and specific online classes such as ‘Zumba’.

*‘Since the diagnosis my partner has been more supportive and is almost dragging me out for walks’*

### Reflective motivation

Focus group participants highlighted that the diagnosis of GDM was an enabler to PA. Women felt it was a prompt to review all aspect of their health and with Pa carrying a higher priority. Finding enjoyable activities that lead to health benefits was also an enabler. Recognition of improvement through self-monitoring and the identification improvement in glycaemic control were specific drivers of behaviour change. However, frustration and lack of physical capacity/ability compared to pre-pregnancy lead some women to avoid PA.

*‘Concerns about the baby and doing everything I can’ &’ it isn’t about me now; it is about my baby’*

*‘Activity is now even more important to my health since the diagnosis of GDM’*

### Automatic motivation

Negative emotions such as fear of discomfort or doing harm thorough PA were common barriers. However, focus group participants highlighted an anxiety of needing to go onto medication as a driver to increase PA. The importance of establishing a routine was also a key enabler.

Table 3 shows the barriers and facilitators mapped on the COM-B and TDF model. The behavioural diagnosis concluded that the following components and domains were most suitable: psychological capability (knowledge/understanding, behaviour regulation), reflective motivation (beliefs about capability and consequences, goals), automatic motivation (emotion), social opportunity (social influences) and physical opportunity (resources).

### Stage 2: identification of intervention options

The four key intervention functions most suitable to address these aspects of COM-B were Enablement (increasing means/reducing barriers to increase capability), Education (increasing knowledge or understanding), Persuasion (using communication to induce positive or negative feelings or stimulate action) and Training (imparting skills). The links between the COM-B model, the TDF and the intervention functions are shown in Table 4. Incentivisation, coercion, environmental restructuring, modelling, and restriction were excluded as they were not considered suitable functions.

**Table 4:** Selection of behaviour sources, intervention function, policy categories, BCTs, intervention strategies/mode of delivery for the intervention developed to promote PA in women with GDM following Motivational interview

Behaviour source targeted in the intervention	Intervention functions	Policy Category	BCTs	App Feature/Mode of delivery
**Psychological capability** women (knowledge) report unaware of PA opportunities)	Education *Information about health consequence* Training: *Instruction on how to perform the behaviour*	Service provision Communication	Information about health consequences (5.1), Credible source (9.1), written persuasion about capabilities (15.1)	Resource centre within the application specific information about the PA & GDM (Hospital Trust leaflet) Exercise booklet on examples of home exercise; promotional posters with a short film of typical activities & health benefits within the resource centre Users are able on the application search for local specific antenatal PA
**Psychological capacity** Behaviour Regulation & Goals	Training: Education Enablement:	Service provision Communication	*Training:* • Self-monitoring of behaviour (2.3) *Education* • prompts/cue (7.1) • feedback on behaviour (2.2) • self-monitoring of behaviour (2.3) •; *Enablement:* • Goal setting (behaviour)(1.1) • Action planning (1.4) • Review behaviour goals (1.5)	Women can set, monitor and review PA Goals with HCP via the application Ability to self-monitor goals via performance wheel Feedback given on performance by automated & personalised message from MI midwife. Reminders & promotional messages Telephone/online weekly review by midwife and plan/adjust weekly goals
**Social Opportunity** Individuals feel self-conscious being active by themselves.	Enablement Environmental restructuring	Communication	Credible source (9.1) Prompts/cues (7.1)	Support from HCP via App/+/− family/partner.
**Physical Opportunity** Environmental context and resources (persons situation or environment)	Enablement	Communication	Instruction to perform the behaviour (4.1.)	Resource centre: Home exercise booklet within the Stay-Active enabling an option for women to fit activity into their lifestyle (learn this skill)
**Reflective motivation**	Education Persuasion	Service provision	Self-monitoring of behaviour (2.3) Credible source (7.1) Written persuasion about capabilities (15.1) Feedback on Behaviour (2.2)	Application Messages encourage/prompt users to reflect on their activity Reflection and feedback from HCP (represent a credible source) as users complete and successfully maintain PA Weekly goals educate/inform users about their PA capabilities When a goal is completed, a positive message is displayed
**Automatic motivation**	Enablement	Communication Service provision	Prompts/cues (7.1)	Regular reminders & promotional messages to prompt a positive habit change/maintenance

Out of the seven policy categories listed in the BCW guide as potentially useful for achieving behavioural change, we identified two:

1. Communication/marketing – for example, using education materials to raise awareness of importance of staying active
2. Service Provision (Establishing support services via the smartphone application).

The other policy categories were excluded as they were considered not appropriate.

### Stage 3: intervention content

Based on the four chosen intervention functions (Education, Enablement, Persuasion and Training), there were a total of 28 listed BCTs which were reported as frequently used, and a total of 63 BCTs reported as less frequently used [34]. Ten selected BCTs were considered appropriate to stakeholders. All other BCTs were excluded. Table 4 shows the mapping of intervention functions, policy categories, BCTs, intervention strategies. Key features agreed amongst stakeholders included goal setting, self-monitoring, performance feedback and motivational messaging. The mode of delivery was via a smartphone application. This mode of delivery had been chosen from the beginning as it provides an easily accessible and multi-functioning tool delivering patient resources and direct messaging. The integrations of the COM-B model and BCW has helped inform this process. The selected BCTs with descriptions and BCT taxonomy are shown in Table 5.

**Table 5:** The Selected BCTs with descriptions

Behaviour Change Technique	BCT description
Goal setting [1.1]	set or agree a goal defined in terms of behaviour to be achieved
Action planning [1.4]	prompt detailed planning of performance of the behaviour must include at least one of the context, frequency, duration and intensity
Review behaviour goals. [1.5]	review behaviour goals (s) jointly with the person and consider modifying goal(s) or behaviour change strategy in light of achievement
Self- monitoring of behaviour [2.3]	Establish a method for a person to monitor and record their behaviour(s)as part of a behaviour change strategy
Instruction to perform the behaviour [4.1]	advice or agree on how to perform behaviour
Credible source [9.1]	present verbal or visual communication from a credible source in favour of or against the behaviour
Written persuasion about capabilities [15.1]	inform the person that they can successfully perform the wanted behaviour
Prompts and cues [7.1]	introduce or define environmental or social stimulus with the purpose of prompting or cueing the behaviour
Feedback on behaviour [2.2*]*	monitor and provide informative or evaluative feedback on performance of the behaviour
Information about health Consequence [5.1]	provide information (e.g. written,verbal, visual) about health consequence

[Bracketed numbers] referred to The Behaviour Change Technique Taxonomy (v1) [42]

### Stay-active

**Fig. 2:**
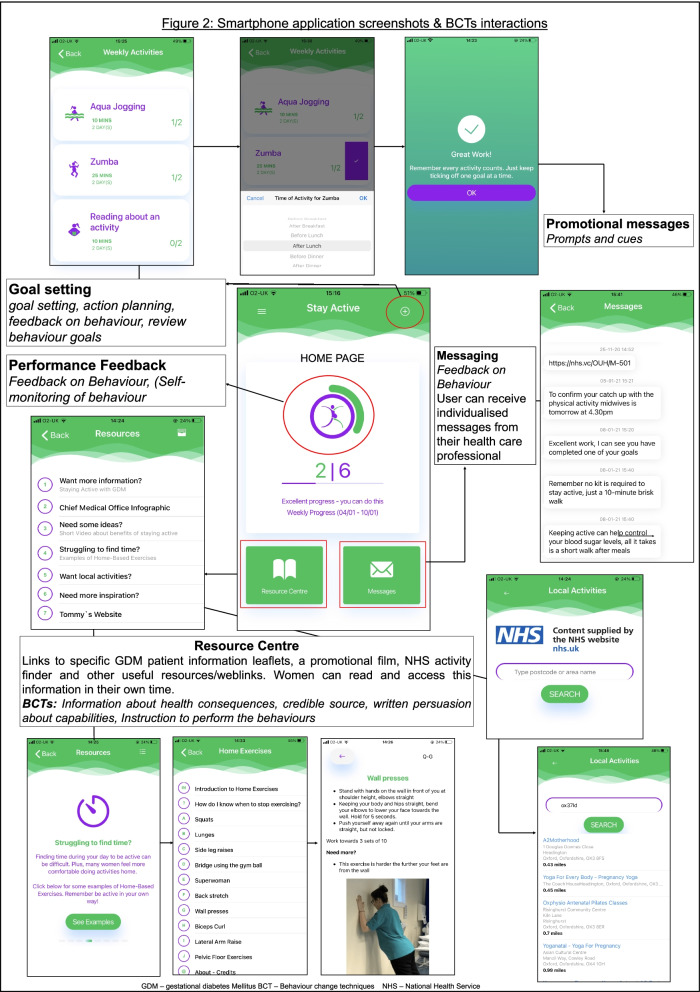
Provides screenshots taken from Stay-Active demonstrating some of the applications features and the selected behaviour change techiques used to support users

The Stay-Active app incorporates four intervention functions and ten BCTs. Stay-Active has been designed to be used in conjunction with the initial motivational interviewing intervention supporting key aspects such as goal setting and feedback. Fig. 2 demonstrates how the BCTs are delivered, and Table 6 highlights how key enablers and barriers to PA are addressed across the motivational interviewing and Stay-Active. At the end of the motivational interview, women will be encouraged to download and setup Stay-Active. A specialist midwife will contact the user on a weekly basis to discuss their progress and support continued behaviour change.

1. **Information about health benefits: Resource centre:**
  *(Information about health consequences, Credible source, written persuasion about capabilities, Instruction to perform the behaviours)*

**Table 6:** Enablers and barriers to physical activity in pregnant women mapped on the motivational interviewing session and Stay-Active

Themes^a^	Motivational Interviewing session	Stay-Active
Information on benefits of PA	Yes	Yes
Information on Types of PA	Yes	Yes
Addressing concerns	Yes	Yes
Provide emotional support	Yes	Yes
Encourage involvement with friend and family	Yes	No
Using prompt or reminder to be physical activity	No	Yes
Accessible resources	No	Yes
Information on resource available in the community (classes for pregnant women)	Yes	Yes
Monitor PA progress	No	Yes
Change social norm (you can be active in pregnancy	Yes	Yes
Time to be physically active	No	No
Home based exercise	Yes	Yes
Supervision/follow input	Yes	Yes

*Abbreviations*: *PA* Physical activity

^a^Themes taken from focus groups, current literature and adapted from Flannery et al. [43]

In time-limited clinical consultations, information and resources about PA compete with other components of care. Key information about PA via Stay-Active is easily and constantly available for women to look through in their own time outside of clinic/appointments. This contains specific resources including a healthcare provider approved leaflet on GDM and PA addressing and explaining specific benefit of PA, an infographic on the benefits and types of PA, examples with explanations of suggested home-based workouts/exercise, a short educational film on the benefits and key messages about PA in pregnancy, an embedded search function for local NHS recommended pregnancy specific PA classes, and links to two credible PA resources (Moving medicine patient information leaflet (https://movingmedicine.ac.uk/) and Tommy’s website https://www.tommys.org/). Aiding to improve knowledge and address barriers such as safety concerns.

2) **Goal setting & action planning**
  *(goal setting, action planning, feedback on behaviour, Review behaviour goals)*

Towards the end of the motivational interview, if appropriate, women are encouraged to set personalised weekly goals with a specialist midwife, usually two or three goals are set for a week. Examples include a brisk walk for 20 min × 3/week or attending a yoga class. Users are able to review and record goals directly onto the application, update and can access them at any time. Their weekly goals are integrated into the performance feedback wheel. Users will be able to ‘sign off’ part or all of their goals on the application as they are completed. There will be a prompt to evaluate their goals and feedback. On a weekly basis, a specialist midwife will contact the user, via telephone, to discuss their progress, current goals and agree adjusted plan if appropriate. Goals can be set remotely via the application (Fig. 2).

3) **Self-Monitoring: **(*Self- monitoring of behaviour*)

The users will be able to record their PA on Stay-Active with tracking of their completed goals on the performance feedback wheel.

4) **Performance feedback:** *(Feedback on Behaviour)*

There is a feedback wheel; as users log their PA (Fig. 2), this will show their personal weekly goal and goal completion percentage. Unique to this application is the ability for the specialist Midwife to interact with the user. The Midwives or HCPs will be able to review the users recorded PA remotely and then directly send users specific tailored messages via the application.

5) **Motivational messages and prompts/cues** *(Prompts and cues)*

When a goal has been completed, users will receive an automated motivational message depending on the stage of completion of their weekly goals.

HCPs can view and monitor their user’s activity progress and communicate feedback by individualised text messages. Users will also receive motivational messages about PA at 10 am every day via the smartphone notification system. These messages were tested and adjusted with our PPI group (Additional file 2 -Table 1).

6) **Tracking activity and Support:**

Specialist Midwives can view how the women progress in real time. HCPs can contact women via the message centre if they have not logged or registered activity. The midwives will provide support over the phone or via the message centre on a weekly basis (Additional file 2).

## Discussion

### Main findings

We describe the systematic development of a smartphone application using the BCW to support PA in women with GDM. This smartphone application has been designed to support and maintain PA alongside the existing successful motivational interviewing intervention. This design includes four intervention functions and ten BCTs delivered via an educational resource, goal setting and action planning features, personalised performance feedback and individualised promotional messages.

Using the BCW facilitated the design to focus on what needed to change for the target behaviour to occur, addressing specific barriers and enablers to support PA. A key aspect is the timing of this intervention, building on a potential ‘teachable moment’ [16] following a diagnosis of GDM where there is opportunity to re-focus on PA with the health of the baby and glycaemic control being strong motivators. Using the BCW helped identify behavioural change components already delivered through the pre-existing motivational interviewing, identify gaps and areas requiring support.

### Comparison to previous literature

Previous studies have evaluated the use of smartphone applications as an adjunct tool to lifestyle intervention during pregnancy with mixed results [44, 45]. A multicentre, nested randomised trial involved 162 pregnant women (10–12-week gestation) [44], whereby 77 women (77/162) in addition to lifestyle advice were provided with access to a smartphone application designed to encourage women to set dietary and PA goals and monitor their progress. Results showed there were no statistically significant differences in PA between the treatment groups. However, only a total of 24 women (31.2%) reported using the smartphone application and there was no mention of any user involvement in development design, or a theoretical framework used to inform the intervention. For our Stay-Active app we hypothesise the addition of the motivational interviewing together with regular follow up and individualised reminder messages, which will maintain engagement over the required period.

Kennelly et al. [45] evaluated the effect of a healthy lifestyle package supported by a smartphone application on the incidence of GDM in 565 overweight and obese women. The intervention consisted of specific dietary and exercise advice that addressed behaviour change, supported by a tailor-designed smartphone application. Self-reported PA levels were measured at baseline and in the third trimester post intervention. Whilst the intervention did not decrease the incidence of GDM, it did result in a greater self-reported exercise participation by the third trimester. This is important because PA tends to decline with advancing gestation.

Garnweidner-Holme et al. designed the Pregnant+ app. The app supports automatic transfer of blood glucose values from the glucometer to the smartphone and also includes information about nutrition and PA for women with GDM [46]. A qualitative study found that women experienced increased confidence in their own GDM-management and increased motivation for behaviour change. The information in the app was considered easily accessible and reliable [47]. However, the results of their multicentre non-blinded randomised controlled trial involving 238 women found the Pregnant+ app had no effect on their primary outcome; 2-h glucose level at routine postpartum OGTT.

Unlike Pregnant+ and other smartphone applications used in pregnancy, our application was designed with specific behavioural change techniques focused only on PA; supporting the initial motivational interview and building on the existing relationship between the HCP and user. This combination is unique to this intervention with features such as individualised and promotional messaging, reviewing goals on a weekly basis which updated in real-time and support from a trusted HCP. The resource centre provides an opportunity for women to review information about PA from a credible source addressing key enablers such as home exercise routines and providing the education on PA types, intensity and specific impact on GDM.

It has been highlighted that technology can have a negative impact. Some women may dislike the idea of tracking PA which is linked to the clinic and it would feel like ‘big brother is watching’ them/an invasion of privacy [43]. However, the relationship built between the user and HCP during the motivational interview will help the user to understand it is a means of providing support. A future merger with a blood glucose monitor application would seek to build on the high levels of patient engagement and allow women to clearly observe the direct impact of PA on their glucose levels.

### Strengths and limitations

This study used an evidence-informed and systematic approach to develop an app to promote PA in women with GDM based on a theoretical framework, the current literature, focus groups and stakeholder engagement. The intervention was specifically designed to meet the needs of our local population and may not be applicable to other regions/healthcare systems. Specifically, it relies on the existing motivational interviewing intervention. Nevertheless, these skills can be transferred and disseminated to other HCPs and could easily be delivered remotely online via video conferencing. It is acknowledged that technology is rapidly advancing and by the time applications are developed, implemented and tested, the technology may be outdated.

The application of the BCW was time consuming [48] but resulted in the suitable selection of intervention functions and BCTs. Whilst the BCW has been applied to the development and evaluation of interventions including modifying PA behaviour [19, 20, 49] it has not previously been applied to the development of interventions to promote PA in women with GDM.

The next steps are to conduct a study of feasibility and acceptability of the combined intervention (Stay-Active + Motivational Interviewing consultation) in women with GDM and evaluating the impact on clinical outcomes, such as objective PA levels and blood glucose measurements. The study protocol is pre-registered with ISRCTN 39136.

## Conclusion

The BCW enabled a systematic and comprehensive development of a novel, multicomponent smartphone intervention to support an existing PA intervention in women with GDM within an NHS Maternity service.

## Supplementary Information

**Additional file 1.**

**Additional file 2.**

## Data Availability

The datasets used and analysed during this current study are available from the corresponding author on reasonable request.
