# Supplementary material for: Applying the behaviour change wheel to develop a smartphone application ‘stay-active’ to increase physical activity in women with gestational diabetes
Source: BMC Pregnancy Childbirth. 2022 Mar 26;22:253. doi: 10.1186/s12884-022-04539-9 (PMC8962081; doi:10.1186/s12884-022-04539-9)
Supplement: Supplementary file 1 — Additional file 1. [file 12884_2022_4539_MOESM1_ESM.docx]

**Additional file 1:**

**Details regarding Existing Motivational interviewing intervention**

Women attending the clinic are invited to engage in a 20-minute individual motivational interview on PA, in addition to their routine care appointments. Most women attending their first clinic appointment are in the third trimester at approximately 28 weeks’ gestation. The motivational interview consultation takes place at their initial hospital appointment following a diagnosis of GDM. It is delivered by a trained healthcare professional (HCP). Each HCP delivering the motivational interview had completed a certificated two-day training course and eight hours of supervised training. The interview is delivered using a framework, where motivational interviewing micro skills (open-ended questions, affirmations, reflections and summaries) are used in all sessions to progress participants through the processes of change (engagement, focusing, evocation, and planning)(1). It includes person-centred goal setting and activity planning if deemed appropriate for that stage of the interview. Specific information about the benefits and types of suggested PA is discussed. Table 1 outlines the structure of the motivational interviewing delivered and the BCTs used.

Results from a published quality improvement project demonstrated encouraging results (2). Self-reported PA levels increased significantly at two-week follow-up, with a mean increase of 75 minutes/week in PA levels and more than half (56%) of the women increasing their activity to meet the PA guidelines(3, 4). The Stay-Active app will seek to build on this initial PA behaviour change supporting women to help maintain their activity levels.

Table 1: Structure of the Motivational interview:

| Part | Details | Behaviour changes techniques: |
| --- | --- | --- |
| 1. Setting the scene & Agreeing the agenda | Establish empathy and rapport and ‘goal congruence’ from the start, (ii)Manage some expectations of the consultation (iii) Give the person a sense of control over the conversation and agreeing the main focus of the conversation |  |
| 2. Exploring a typical day | Understanding of a particular aspect of the patient’s life, where activity fits into their lifestyle (ii)Demonstrates non-judgemental, person-centred listening skills. (iii) Listen for any ‘change-talk’ -indicating that the patient is thinking about change, wants to change, is able to change, has already started to make some changes, etc. (iv)Help them feel heard and understood |  |
| 3. Exploring importance | (i)Explore the importance of activity and their reasons for changing their activity levels (ii)Help the person give voice to, and better understand their own reasons for changing (iii) Elicit and develop change talk (iv)Strengthen the other persons readiness to change | Prompt and cues 7.1 |
| 4. Sharing information on benefits | Ask -Share-Ask information about benefits of physical activity specifically for GDM | Information about Health Consequence 5.1  Credible source 9.1 |
| 5. Sharing specific information/knowledge about activity | Ask -Share-Ask information about type, during and expectations about physical specifically for GDM, Address barriers about activity, discuss type, time, frequency | Information about Health Consequence 5.1  Instruction on how to perform behaviour 4.1  Credible source 9.1 |
| 6. Exploring and building confidence | (i) Strengthen their self-efficacy for change.(ii) Elicit and develop change talk.(iii) Share with them what other people have found helpful when making the change e.g. glucose control (using ask-share-ask) | Prompts and cues 7.1  Focus on past success 15.3 |
| 7. Sharing info about building confidence | Ask -Share-Ask information about increasing confidence to become more active (ii) Provide suggestions, increase readiness | Valued self-identity 13.4  Social comparison 6.2 |
| 9. The Key question | Help the person decide what to do next | Problem solving 1.2 |
| 10. Exploring options | (i)Generate a range of possible ways forward (ii)Build optimism and confidence that change is possible.  Encourage autonomy and personal decision making.  Share some of your experience and expertise about what might be helpful (ii) Make progress towards agreeing the way forwards. | Social support 3.1 |
| 11. Agreeing a plan and goal setting | Help the person generate a plan for their future (ii)Help Evoke ideas (iii) Complete personal Goal setting tool | Goal setting (behaviour) 1.1  Action planning 1.4 |
| 12. Relapse prevention | Help the person explore how their life might be different if they did decide to (and were able to) change, compared to if they didn’t. (ii) Help the person better understand the risks of not changing and the benefits of changing, without you having to tell them (iii) ‘Develop discrepancy’ between their current behaviour and their desired future behaviour (iv) Learn more about the persons hopes, plans and values (v)Build hope Elicit and develop change talk. (vi)Agree about the need and timing of future conversations (vii)Agree about the medium and location of future conversations –face to face, telephone | Comparative imaging of future 9.3  Verbal persuasion about capability 15.1  Commitment 1.9 |
| 13. Support | Explaining the support offered. | Social Support 3.1 |

The numbers in brackets related to the code for the behaviour Change Technique (BCT) as per the Behaviour Change Technique Taxonomy Version 1.(5)

‘The framework and content of the motivational interview (table 1) was developed with the support and assistance from the Academy for Health Coaching

<https://learn.academyforhealthcoaching.co.uk/>

1. Miller WR, Rollnick S. Motivational interviewing : helping people change. 3rd ed. ed. New York, NY: Guilford Press; 2013.

2. Smith R, Ridout A, Livingstone A, Wango N, Kenworthy Y, Barlett K, et al. Motivational interviewing to increase physical activity in women with gestational diabetes. British Journal of Midwifery. 2021;29(10):550-6.

3. Mottola MF, Davenport MH, Ruchat SM, Davies GA, Poitras VJ, Gray CE, et al. 2019 Canadian guideline for physical activity throughout pregnancy. Br J Sports Med. 2018;52(21):1339-46.

4. Dipietro L, Evenson KR, Bloodgood B, Sprow K, Troiano RP, Piercy KL, et al. Benefits of Physical Activity during Pregnancy and Postpartum: An Umbrella Review. Med Sci Sports Exerc. 2019;51(6):1292-302.

5. Michie S, Richardson M, Johnston M, Abraham C, Francis J, Hardeman W, et al. The behavior change technique taxonomy (v1) of 93 hierarchically clustered techniques: building an international consensus for the reporting of behavior change interventions. Ann Behav Med. 2013;46(1):81-95.
