# Supplementary material for: Applying the behaviour change wheel to develop a smartphone application ‘stay-active’ to increase physical activity in women with gestational diabetes
Source: BMC Pregnancy Childbirth. 2022 Mar 26;22:253. doi: 10.1186/s12884-022-04539-9 (PMC8962081; doi:10.1186/s12884-022-04539-9)
Supplement: Supplementary file 2 — Additional file 2. [file 12884_2022_4539_MOESM2_ESM.docx]

**Additional file 2:**

Midwives can view their patient’s activity progress, communicate by sending individualised feedback messages and adjust weekly goal setting remote:

Figure 1:


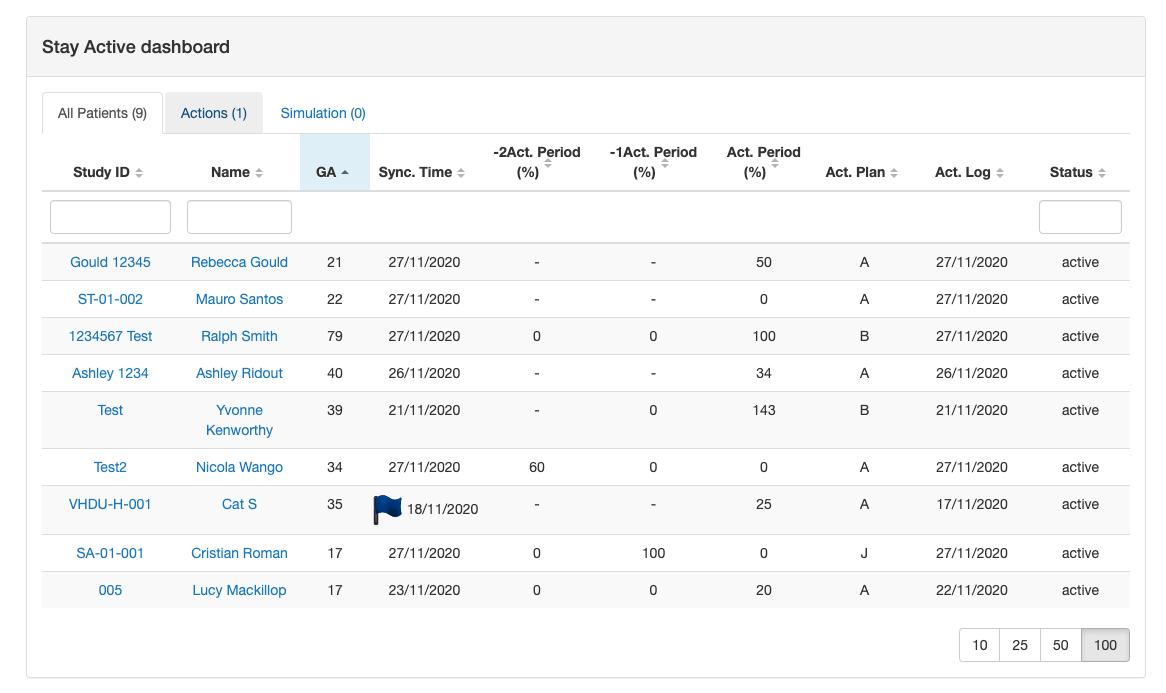


**Table 1: Motivational messages:**

| Daily Notification messages | 1. Staying active helps boast energy levels throughout the day and you’ll get a better night’s sleep. 2. Keeping active can help control your blood sugar levels, all it takes is a short walk after meals (more information – link to GDM patient leaflet) 3. A brisk walk after meals can help keep your blood sugar levels in check 4. Keeping active isn’t easy in pregnancy, don’t pressure too much on yourself, be active in your own way 5. Being active can improve your mood & sense of well-being. 6. Remember no kit is required to stay active, just a 10-minute brisk walk 7. Activity/exercise releases feel-good hormones that improve your mental wellbeing |
| --- | --- |
| Post completion of goal: | 1. Congratulations, well done for completing part of your weekly goal. Staying active is key part of your treatment and help control your blood sugar levels. 2. Great work! Remember every activity counts. Just keep ticking off one goal at a time 3. Fantastic, one another goal down, just take one at a time. Staying active isn’t easy but it is key part of your treatment (see link to leaflet) 4. Congratulation, you’re doing really well, keep active in your own way. 5. Congratulations! keep working towards your goals! 6. Brilliant work- keep moving for you and your baby 7. Well done, you’re making great progress for you and your baby. 8. Congratulations, another goal down! 9. Excellent work, staying active can help manage your sugar levels. 10. Fantastic effort, keep progressing with your goals |
